# “Optical communication with brain cells by means of an implanted duplex micro-device with optogenetics and Ca^2+^ fluoroimaging”

**DOI:** 10.1038/srep21247

**Published:** 2016-02-16

**Authors:** Takuma Kobayashi, Makito Haruta, Kiyotaka Sasagawa, Miho Matsumata, Kawori Eizumi, Chikara Kitsumoto, Mayumi Motoyama, Yasuyo Maezawa, Yasumi Ohta, Toshihiko Noda, Takashi Tokuda, Yasuyuki Ishikawa, Jun Ohta

**Affiliations:** 1 Laboratory for Developmental Gene Regulation, Brain Science Institute, RIKEN, Wako, Saitama 351-0198, Japan; 2 Graduate School of Materials Science, Nara Institute of Science and Technology, Ikoma, Nara 630-0192, Japan; 3 Department of Systems Life Engineering, Maebashi Institute of Technology, Maebashi, Gunma 371-0816, Japan

## Abstract

To better understand the brain function based on neural activity, a minimally invasive analysis technology in a freely moving animal is necessary. Such technology would provide new knowledge in neuroscience and contribute to regenerative medical techniques and prosthetics care. An application that combines optogenetics for voluntarily stimulating nerves, imaging to visualize neural activity, and a wearable micro-instrument for implantation into the brain could meet the abovementioned demand. To this end, a micro-device that can be applied to the brain less invasively and a system for controlling the device has been newly developed in this study. Since the novel implantable device has dual LEDs and a CMOS image sensor, photostimulation and fluorescence imaging can be performed simultaneously. The device enables bidirectional communication with the brain by means of light. In the present study, the device was evaluated in an *in vitro* experiment using a new on-chip 3D neuroculture with an extracellular matrix gel and an *in vivo* experiment involving regenerative medical transplantation and gene delivery to the brain by using both photosensitive channel and fluorescent Ca^2+^ indicator. The device succeeded in activating cells locally by selective photostimulation, and the physiological Ca^2+^ dynamics of neural cells were visualized simultaneously by fluorescence imaging.

Understanding the functional neural cells activities in the brain that are related to psychological and physical activities is one of the most important issues in neuroscience today. Noninvasive optical methods are useful, powerful tools for functional brain analysis, because such methods enable wide-ranging analyses with high spatiotemporal resolution without destroying tissue. A number of such tools have recently been developed. Optogenetics is spatially and temporally precise, which allows specific cells of living tissue to be selectively targeted^1^,^2^,^3^. A gene of a photosensitive channel protein confers light responsiveness to the transfected cell. In other words, the genetically encoded switches allow neurons to be turned on or off with light of certain wavelengths. In addition, neural activity can be stably visualized in broad areas using a genetically encoded Ca^2+^ indicator, which shows the intracellular calcium status as changes in fluorescence intensity^4^,^5^,^6^. Such an indicator permits constant long-term imaging without quenching, drift, or reloading upon every measurement, unlike dye-type indicators. Several instruments that photostimulate neurons in the brain with optogenetics have recently been developed^7^,^8^,^9^, in addition to several functional brain-imaging techniques^10^,^11^,^12^,^13^,^14^. Micro complementary-metal-oxide-semiconductor (CMOS) image sensors enable less invasive imaging in living tissue^15^,^16^,^17^,^18^,^19^,^20^,^21^. Previous studies have demonstrated that a fluorescence imaging system enables potentiometry in primary cultured neurons and in the brain with multiple sensors^22^,^23^,^24^,^25^. These compact instruments for functional brain measurements in a freely moving animal, incorporating optogenetics and Ca^2+^ imaging, will provide insight into the natural behavior of animals. Such a technique would be useful for functional brain analysis and could be used to control perception, behavior, and emotion in a freely moving animal. As such, optical neural interface devices have been investigated heretofore^26^,^27^,^28^,^29^,^30^,^31^. In the present study, an implantable device incorporating optogenetics and Ca^2+^ imaging and its control system was newly developed. The device has eight green light emitting diodes (LEDs) uniformly distributed around a CMOS chip for fluorescence excitation, three separate blue LEDs for localized photostimulation, and a CMOS sensor chip for Ca^2+^ imaging (Fig. 1b). The lights for stimulation, excitation, and emission do not mix because their wavelengths are different (Fig. 1a). To the best of our knowledge, this is the first report of an implantable large-scale integration (LSI)-based CMOS device that can perform both photostimulation and optical imaging of neurons in the brain simultaneously, using both a photosensitive channel gene and a fluorescent physiological indicator gene.

## Results

### Validation of bidirectional, optical communication with a cell

We firstly tested whether it is feasible to stimulate a cell with blue light and simultaneously visualize its physiological state via intracellular Ca^2+^ kinetics as a change of red fluorescence intensity using green excitation light. The Neuro2a cell line was chosen rather than crude primary cultured neural cells because it was expected that Neuro2a cells would provide a uniform reaction. Neuro2a cells co-transfected with ChR2-Venus and R-GECO1 were prepared and plated on a glass bottom dish. ChR2 in transfected cells can be visualized by Venus fusion of a green fluorescent protein (Fig. 2a). Although green fluorescence derived from Venus was invariant, R-GECO1 is dim in the absence of Ca^2+^ and bright when bound to Ca^2+^, which leads to an increase in red fluorescence due to the increase in the intracellular Ca^2+^ concentration. Accordingly, blue light stimuli activate ChR2, open cation channel gates, cause depolarization via Ca^2+^ inflow, and increase red fluorescence. The fluorescence image was taken using an inverted fluorescence microscope (DMI 6000 B, Leica Microsystems Inc., Germany) and a digital camera (ORCA-R2, Hamamatsu Photonics Inc., Japan). Transient blue light was subsequently applied to the cell during intermittent imaging of the red fluorescence with green excitation lighting (Fig. 2b). As a result, the red fluorescence intensity increased transiently, as judged from regions of interest (ROIs) whose changes were analyzed with ImageJ (Fig. 2c). *In vitro* experiments have shown that R-GECO1 exhibits a 1600% change in intensity^6^. The change in intensity was slightly higher in our experiment. These results demonstrated that optogenetic blue light stimulation was compatible and feasible with red fluorescence Ca^2+^ imaging by green light excitation.

### On-chip 3D culture of Neuro2a cells with extracellular matrix gel

Neuro2a cells generate neurites like neurons when cultured with a special medium for differentiation. We firstly validated what kind of medium was preferable for differentiation of Neuro2a cells (Fig. 3a). DMEM was used as the base medium. No. 1 included 10% FBS, No. 2 included 1% FBS, No. 3 included 1% FBS and 1% dimethyl sulfoxide (DMSO), No. 4 included 1% FBS and 15 mM CaCl_2_, No. 5 included 1% FBS, 1% DMSO, and 15 mM CaCl_2_, No. 6 included 1% FBS and 20 μM retinoic acid (RA), and No. 7 included 1% FBS, 1% DMSO, 15 mM CaCl_2_, and 20 μM RA (see Table S1 in supplementary information). After culture for 3 days, the differentiative medium was changed to Neurobasal Medium with B-27 supplement (Life Technologies, Inc., USA). No. 1 was normal and undifferentiative medium. No. 2 was a medium having a low proliferative condition that is used, for example, to cause transdifferentiation. In this experiment, Nos. 1 and 2 were used as negative controls. Nos. 3, 4 and 6 were reported previously for Neuro2a cell differentiation^32^,^33^. No. 5, a mixture of Nos. 3, 4 and 7, a mixture of Nos. 3, 4 and 6, are both new conditions. After culture for 5 days, total cell number as a result of cell proliferation was automatically counted using ImageJ software in a bright-field image to assess the proliferation potency. Because it was difficult to discern the cell that was the origin of individual neurites under confluent conditions, the differentiated cells were visually identified judging from the fluorescence image of cells that were transfected in advance with green fluorescent protein. As a result, the total number of Neuro2a cells cultured with each medium showed values opposite those of differentiated cells, which is reasonable. Predictably, No. 5 including both DMSO and CaCl_2_ increased the differentiated cell rate more than Nos. 3 and 4 individually. Surprisingly, No. 7, which included DMSO, CaCl_2_, and RA, was expected to have the highest rate of differentiated cells, but its actual result was less than that of Nos. 5 and 6. However, No. 7 has a presumptive total differentiated cell number, which is [the differentiated rate] × [total cell number] (=240) more than No. 5 (=237) and No. 6 (=97). In conclusion, cells in No. 7 maintain a higher proliferative capacity than in Nos. 5 and 6, and have a higher rate of differentiation than Nos. 1–4. In fact, more cells differentiated in No. 7 than in Nos. 5 and 6. These results indicate similar *in vivo* neurogenesis whereby progenitor cells differentiate progressively to form a self-renewing cortical layer. Therefore, it is thought that No. 7 is better than No. 6, in which almost all cells differentiate early in the culture at once. There are no reports that Neuro2a cells can be differentiated with the known condition of No. 3, 4 or 6 to show any physiological activity like a mature neuron. DMEM including CaCl_2_ is a basal culture medium. Thus, the slight differentiation in No. 2 is likely affected by Ca^2+^, which suggests that cells in No. 3 were already under the influence of a little Ca^2+^. For this reason, Neuro2a cells were expected to differentiate to mature nerve-like cells with No. 7 but not with No. 5.

Next, to form an artificial neural network similar to that in the brain sterically, three-dimensional (3D) culture was performed with No. 7 medium. Extension of neurites is known to be affected by ambient environmental conditions. In particular, the extracellular matrix, including collagen, is known to anchor neural cells^34^,^35^. Therefore, the influence on neurite extension by the difference in the hardness and type of extracellular matrix was investigated (Fig. 3b). Results show that Type I collagen-gel did not promote neurite outgrowth regardless of the difference in hardness, while ECM-gel did.

Based on the abovementioned results, the 3D culture of Neuro2a cells was performed on the semiconductor chip with No. 7 medium and ECM-gel as the optimum differentiation culture condition (Fig. 3c), and neurite expansion and 3D construction were successful.

### Utility test of photostimulation and fluoroimaging system

The 3D neural cell culture acts as a brain phantom and will be helpful in artificial neural network analysis and tissue reconstruction in regenerative engineering. The on-chip 3D culture condition was examined preliminarily with a view toward real-world applications. To verify whether the created system was capable of photostimulating cells and capturing an image of the change in fluorescence intensity simultaneously, a 3D culture of Neuro2a cells on the sensor was analyzed *in vitro* (Fig. 4a). Neuro2a cells transfected with ChR2-Venus and R-GECO1 were confirmed using a fluorescence stereomicroscope (M165 FC, Leica Microsystems Inc., Germany), each of them were visualized as green or red fluorescence (Fig. 4c,d), and a red fluorescence image was captured by the sensor (Fig. 4e). These images revealed that cells covered almost the entire sensor surface. Next, Neuro2a cells were stimulated by the blue LEDs during fluorescence imaging with the green LEDs (see Fig. S2 for experimental workflow). A pseudo-colored image shows the change in the fluorescence intensity after the stimulation (Fig. 4f), and time-lapse images at the position indicated in Fig. 4f (black square) are shown in Fig. 4g. The sensor was driven at 27.5 ms/frame. The favorable frames are shown regardless of temporal axes, and the white frames indicate a moment of photostimulation. According to the real-time monitoring of the cell activities, the subsequent stimulation was applied serially after the activations of the cells became calm. The measured values of the fluorescence intensity at the ROIs are shown as a graph in Fig. 4h. The some noise signal in Fig. 4h would be mainly caused by the part of analog circuit from the transit board to the control board. Cells are thought to be present in ROIs 1 and 2 because the fluorescence intensity changes remarkably, whereas ROIs 3 and 4 are considered not to contain cells because there is almost no change in fluorescence intensity. In Fig. 4h, the blue line indicates the mean of the actual values of the fluorescence intensity in ROIs 3 and 4 subtracted from that in ROIs 1 and 2. The magenta line indicates the mean for 50 frames of blue line data. The red bars and arrows indicate application of the blue light stimulus. Judging from the magenta line, the first and second short stimuli of 1.35 and 1.84 s cause slight increases in fluorescence, whereas the third and fourth long stimuli of 11.2 and 11.9 s cause large, graded increases. In addition, the increased fluorescence intensity decreases to near baseline after a short time. These data indicate that the sensor succeeded in visualizing the intracellular Ca^2+^ status and transient stimulation with light simultaneously, which means that the sensor can be used for optical communication with neural cells.

It was subsequently verified whether Ca^2+^ influent can be induced focally by the blue LEDs in different locations on the device (Fig. 4i; left/upper/right position). After photostimulation for approximately 10 s which is enough to get a strong change of fluorescence intensity in Fig 4h, cell activation occurred depending on the LED position. On the other hand, Neuro2a cells transfected with R-GECO1 only or stained with red fluorescent dye (PKH26, Sigma-Aldrich, USA) did not respond to the same photostimulation (Fig. 4k,l). These results indicate that the sensor is able to conduct spatiotemporally specific stimulation of the target cell, and that the Ca^2+^ inflow in Neuro2a cells occurs depending on blue light stimulation only.

### Bidirectional light signal transmission in the brain by the implanted photostimulation and fluoroimaging sensor

To verify whether our system works as expected in the brain, as it does *in vitro*, an *in vivo* experiment was performed in the mouse visual cortex using the sensor and Neuro2a cells transfected with ChR2 and R-GECO1 (Fig. 5a,b). Similar to an application method used in regenerative therapy, a trimmed cell sheet made by 3D culture was inserted interstitially under the open dura and was grafted to the cortical surface that was exfoliated beforehand (Fig. 5c), and a cell clump that was prepared by aggregation on a 1% agarose gel bed was implanted into the cortex (Fig. 5d). All manipulations were performed gently and meticulously using a glass capillary and a sharpened tungsten needle knife in a manner similar to that described previously^36^,^37^. To prevent an acute immune rejection, 200 μl of 0.05% cyclosporine was injected prophylactically into the peritoneal cavity of the mouse before the implantation. After confirming the implanted position by fluorescence stereomicroscopy, the sensor was applied to the cortex. Generally, the animal’s head is strictly fixed during fluorescence-imaging microscopy, and the use of an artificial heart-lung apparatus and an anti-vibration table to reduce noise is preferable. Fortunately, the applied CMOS sensor moves in conjunction with the breathing and heartbeat of the host mouse. This makes measurement simple and easy, which are advantages the microscopy. In a manner similar to the *in vitro* experiment, the cortex was photostimulated by the blue LEDs during the fluorescence imaging with the green LEDs. As a result, implanted cells were activated based on the blue light (Fig. 5e,f). The fluorescence intensity increased just after stimulation at the position of the cell sheet and cell clump and then decreased gradually. These data indicate that the sensor is capable of *in vivo* photostimulation and functional cell imaging.

### Optical communication in the brain using the implantable device

Finally, to demonstrate the validity and usefulness of our new methodology, the device was applied in a mouse whose cortex was co-transfected with ChR2 and R-GECO1 using in utero electroporation (see Methods for details). However, the physiological activity of the neural cells could not be detected clearly by our device. It was thought that real neural cells transfected with R-GECO1 might exhibit only a small change in fluorescence intensity because of uncertain various causes; for example, *in vitro* experiment has good signal/noise ratio while *in vivo* experiment has bad signal/noise ratio because the neural cells exist in deep brain and the signal is inhibited by the extra cellular matrix and myelination *in vivo*. Therefore, we obtained other fluorescence indicators that were expected to have a larger rate of change, and they were screened using human cervical carcinoma HeLa cells, which have been successfully used as a model system in Ca^2+^ signal studies (Fig. S3). It is particularly important to determine which indicator is preferable for imaging with our device. Therefore, the detection ability of the device was inspected using an *in vitro* micro-incurrent evaluation system with a medicinal solution that was newly constructed with the selected candidate indicators (Fig. S4). As a result of these experiments, O-GECO1 was selected as the best indicator of the fluorescence intensity change. Then, ChR2 and O-GECO1 were co-electroporated into the mouse cortex. It was confirmed beforehand that green- and red-fluorescent proteins were detectable on the cortex of a postnatal day 14 mouse when ChR2 and O-GECO1 were co-electroporated to the cortex on embryonic day 16 (Fig. 6a–c). The mouse was set on the lab-made stereotaxic instrument for pups, the device was applied to the cortex after the craniotomy, and both the optical stimulation and optical analysis were simultaneously performed using the device (Fig. 6d). When the mouse cortex was stimulated locally by one blue LED on the right side of the sensor, the fluorescence intensity increased at the same side of the local stimulation and at the portion where the fluorescent cells were present, as judged from comparisons with the fluorescence image taken by the stereo microscope. In contrast, the signal neither increased at locations without fluorescent cells, nor at locations far from the blue LED light (Fig. 6e–h). Corresponding to four bursts of light (50 Hz each, 1, 5, 5, and 10 min), the signal within ROIs 1 and 2 increased. However, the signal of ROI 3 seemed to reach a plateau and gradually decay, although it slightly increased just after the fourth stimulation (Fig. 6h).

Although blood flow changes were often detected, such intrinsic signals usually appear as a dynamic creeping pattern and are basically larger than cellular size. For example, it is visible in the upper side of the images of Fig. 5e. Consequently, it is concluded that the signal islands of cellular size that responded to blue light stimulation were actually via real neural cells. This suggests that the bidirectional, optical communication with neural cells in the brain using the device was successful. Because O-GECO1-expressing cells also express ChR2 simultaneously in this experiment, the fluorescence response in optically stimulated cells was observed. However, we do not deny the possibility that the observed activities of the cells were indirectly enhanced and/or attenuated conversely to confine the propagation of the excessive activity, like an excitatory or inhibitory neuron does, as a result of an artificially activated cell stimulating other cells.

## Discussion

In this paper, an implantable device and a gene transferred mouse capable of transmitting an optical signal bidirectionally were newly developed, and optical transmission was performed in a living animal brain. As shown in Figs 4 and 6, especially in Fig. 4h, the cell responded to photostimulation while the physiological status of the cell was determined using light (fluorescence). At that time, the experimenter could precisely apply the consequent stimuli in read-time when the fluorescence intensity of the cell was decreased, repeatedly. These results indicate that the experimenter actually did communicate with the cell using light voluntarily. Therefore, we can represent that optical communication with brain cells by means of an implantable device was successful. Such an optical method enables localized stimulation and a spatially precise measurement, which are nearly impossible using conventional electrophysiology. Additionally, it may evade the problem that a metal electrode corrodes and is ruined to be covered with such as glial cells, which are facilitated to acquire the migratory property toward the electrode with electric field^38^,^39^,^40^, by *in vivo* long term use.

We made the devices that were two different variations of the setting place of blue LED (Fig. S1). These devices have the same ability to perform the fluorescence imaging. The device of type A (Fig. S1a) was used in Fig. 5, while the device of type B (Fig. S1b) was used in Figs 4 and 6. It was suspected that the different responses by the stimulation from each blue LEDs could not be distinguished clearly because the place of blue LED s are near each other. However actually, the different responses by the stimulation from each blue LEDs could be distinguished without a problem (Fig. 4j).

Reactions, such as propagative neural activities or transient spikes, are not detected currently in the experiment shown in Fig. 4. Each medium used in this study did not seem sufficient for Neuro2a cells to differentiate to a mature neuron. Leastwise, using primary cultured neural cells instead of Neuro2a cells, it would be possible to research more complicated neural networks using a similar method in the future.

Concerning the absorbent filter, the sensor has a tradeoff for optimum performance between the shielding property against blue light stimulation for sufficient ChR2 activation and the perviousness for slight variations in red fluorescence. In other words, it is difficult for the selected filter to detect small changes in fluorescence during photostimulation (whiteout frames in Fig. 4g), which is due in part to the wide spectrum edge of the LED. To visualize the response during photostimulation, it is preferable to narrow the spectrum edge of the LED and/or further separate the wavelength area of the blue and red lights. In fact, this type of excitation filter for the LED and an infrared fluorescence indicator would be useful.

In the past experiment, the image sensor could be able to receive the illumination from the different area onto the whole photo-diode array^24^. However, it is not able to avoid that a slight fluorescence incline occurs. For example, the fluorescence intensity of under position in the image of Fig. 4e is slightly darker than that of upper position. In the future, the fluorescence incline should be corrected rigorously using the mathematical approach if needed.

Bloodstream changes were clearly detectable in the living brain, which sometimes disturbed the fluorescence analysis (Fig. 5e,f). For efficient fluorescence imaging, it is preferable to use a “biological optical window”, of which the spectral range is approximately 650 to 1,200 nm, in order to avoid the high absorption range of hemoglobin and water^41^,^42^,^43^,^44^.

In Fig. 5a and 6e, the device was put on the brain surface. When the device is applied for the awake mouse that is freely moving, the cranial bone piece is returned to cover the device that is put on the brain surface after the craniotomy. Then, the cranial bone and the device were fixed using surgical adhesive and dental cement. Finally, the wound of the scalp is sewed up, and the implantation operation of the device is completed^24^. In the similar manner, the device can be used for not only the application on the brain surface but also the deep brain imaging^17^,^23^.

Mice has a flattened cortex, thus, the image sensor chip of the small size can be attached to the parietal cortical area with a coherent contact. To image broad area with the coherent contact on the curved brain surface, it is necessary to use several sensor chips combined with each other^25^. In the future, the many sensor chips would be able to be applied to the complex curved brain in combination with a more flexible substrate^45^.

The stimulation range of a single blue LED appeared to be approximately 300 μm from the LED chip (Fig. 4j). The range size is similar to the estimated functional unit, the cortical column, in monkey visual cortex^46^,^47^. Therefore, the present sensor would be more suitable for large animals, such as monkeys and humans, to stimulate each column individually. Miniaturizing the LED chip, modulating the strength of the LED light, and driving the photosensitive gene under a spatiotemporal specific promoter would help to stimulate each column more individually.

Although the proposed system contains some sophisticated refinements, this system is compact and has superior portability. Unlike bulky fluorescence microscope systems that use solid-state lasers, the proposed system has independent photostimulation, excitation, and fluorescence imaging capabilities without the need for extra equipment. As such, animals implanted with the proposed device can move freely. In addition, the proposed method can be applied to the entire brain using multiple LEDs and sensors arranged in a mosaic pattern with a flexible web-like form that can be controlled simultaneously^25^. It is also possible to use wireless transmission^48^. These advantages would be useful in a perception prosthesis technology or a brain-machine interface. In the future, it is hoped that the proposed methodology will be applied to artificial vision techniques that involve brain stimulation in conjunction with gene therapy using an adeno-associated virus, which is a candidate for clinical application^49^.

Finally, we conclude that a system for optical functional analysis of the brain was constructed. The system drives a micro-implantable device that enables selective photostimulation and a fluoroimaging sensor that was developed using CMOS technology. The present paper demonstrated that the system was capable of stimulating photosensitive cells and visualizing the physiological activity of the cells simultaneously both *in vitro* and *in vivo*. This type of implantable, bidirectional, optical neurocommunicator is strongly desired because such a device will be useful in the evaluation of neural propagation depending on artificial stimulation and appropriate inhibition of neural activity depending on the detection of abnormal neural activity in a freely moving individual. Such a device would also be useful for medical care and contribute to advances in neural prosthetics.

## Methods

### Animal studies

All procedures involving animals were approved by RIKEN committees (Wako Animal Experiments Committee and Genetic Recombinant Experiment Safety Committee) and the Nara Institute of Science and Technology (NAIST) Animal committees, and were performed in accordance with the institutional guidelines of the animal facilities of RIKEN-Brain Science Institute (BSI) and NAIST. C57BL/6J and ICR mice (8 weeks old, pregnant mice, SLC Co., Japan) were used for *in vivo* experiments. A mouse, anesthetized by intraperitoneal injection of 20 mg/ml Avertin (2, 2, 2-Tribromoethanol) (0.25–0.5 mg/g body weight), was mounted on a stereotaxic instrument (Narishige Co., Japan) or a laboratory-made brain stereotaxic apparatus for pups (Fig. 6d). After craniotomy using a dental drill, graft implantation and sensor application were performed. Image data were analyzed using ImageJ (supplied by the National Institute of Health, USA).

For in utero electroporation, ICR mice at approximately embryonic day 16 were used because the neurons on the top of the cortical plate, approximately layer II–III neurons of the cortex, are generated at these stages^50^. After laparotomy of the anesthetized pregnant mice, DNA plasmid solution was injected into the cerebral ventricle of the embryo using a glass capillary through the uterine wall. Electric pulses were subsequently applied to the embryos by gently clasping their heads with electrodes using a square-pulse generator (CUY21EDIT, BEX Co., Japan).

**Figure 1:**
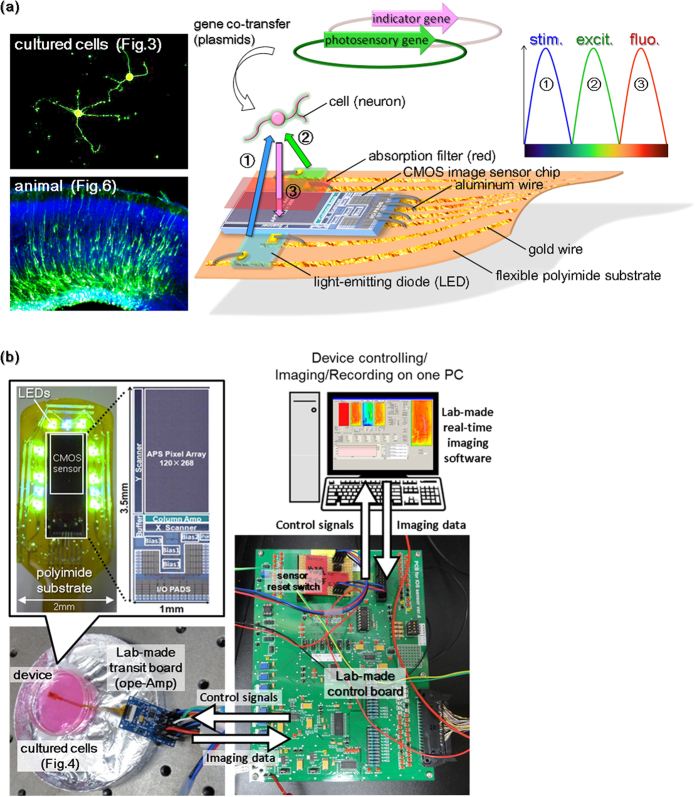
(**a**) Schematic diagram of a concept for bidirectional, optical neurocommunication. After gene-transfer of the photosensory gene and fluo-indicator gene into the cell, (**1**) the cell was stimulated by a blue LED and (**2**) excited by a green LED. (**3**) The dynamic change in red fluorescence emitted from the cell, which passed through an absorption filter, was imaged using a CMOS sensor. The fluorescence image labeled “cultured cells” shows Neuro2a cells, which were differentiated and matured to form neurites by long-term culture (see Fig. 3 for details), and “animal” is the frozen section of the mouse brain at the visual cortex region stained with DAPI. Gene-transfer in the mouse was accomplished using in utero electroporation (see Fig. 6 for details). (**b**) This diagram shows the control system of the bidirectional light transmission device. The device is connected to a control board through a transit board and is controlled by a single PC.

**Figure 2:**
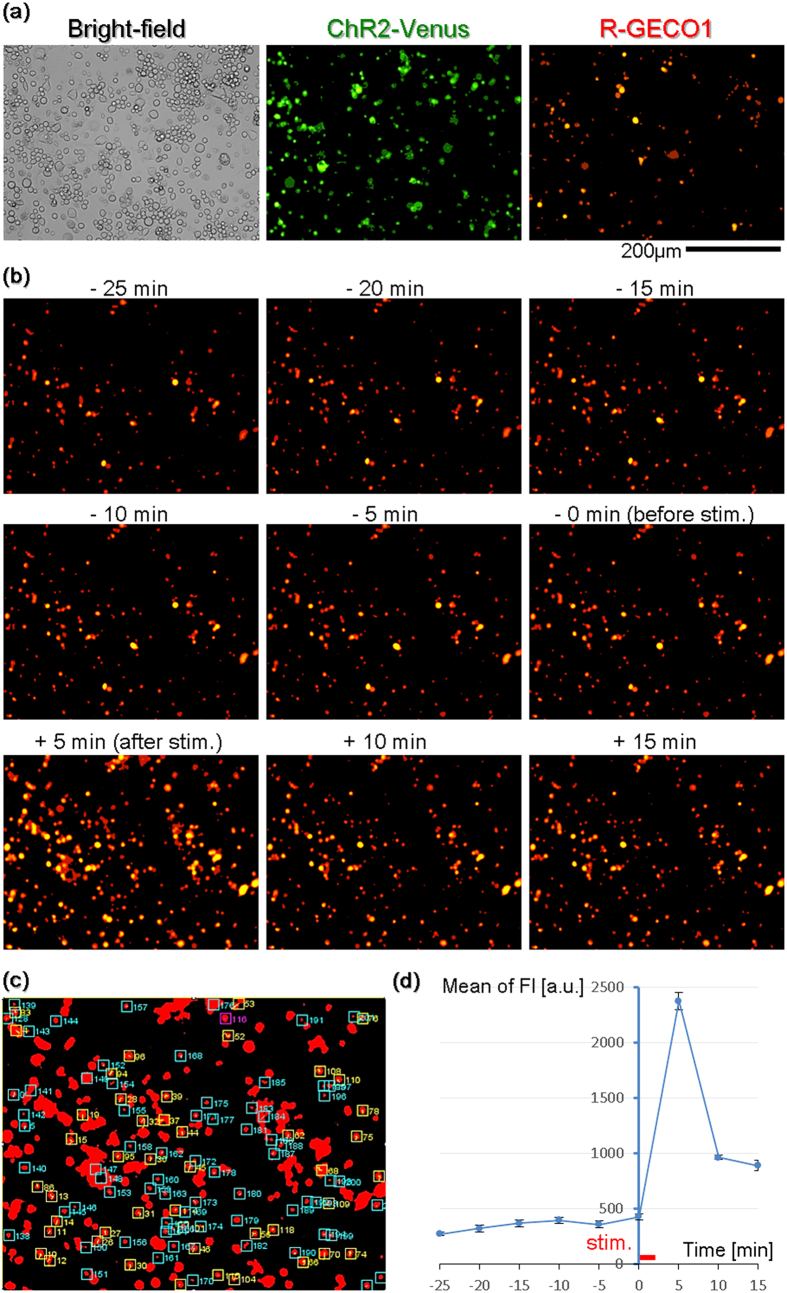
Confirmation of the bidirectional, optical communication with the cultured cell. (**a**) Bright-field and fluorescence images of Neuro2a cells that were co-transfected with ChR2-Venus and R-GECO1 on the glass bottom dish were taken with an inverted fluorescence microscope. (**b**) Neuro2a cells were stimulated by transient blue light for 2 s while the red fluorescent images were continuously taken. (**c**) ROIs of (**b**) are shown. A total of 276 ROIs were analyzed, and then 47 ROIs that showed a clear response were extracted (yellow). The top six ROIs indicating a large response were extracted from yellow ROIs, and the values are plotted in the graph [blue line in (**e**)]. The 116 magenta-colored ROIs indicate areas with the highest dF/F value (2053.66%).

For fixation, postnatal mice were deeply anesthetized and perfused transcardially with ice-cold physiological saline, 4% paraformaldehyde (PFA) in 0.1 M phosphate buffer (PB). After the brains were isolated, they were further fixed with 4% PFA/PB overnight at 4 °C and then immersed in 30% sucrose/PB overnight at 4 °C. The brains were frozen rapidly by dry ice powder and then cryopreserved. Frozen serial sections were cut using a cryostat, mounted onto Matsunami adhesive silane-coated glass slides, stained with DAPI (4′,6-diamidino-2-phenylindole) to visualize the nuclei, and then observed by fluorescence microscopy (Fig. 1a).

### Design and development of the photostimulation and fluoroimaging system

The image sensor was produced using a 0.35-μm 2-poly 4-metal standard CMOS process by Austria Micro Systems, based on a three-transistor active pixel sensor^51^,^52^. The design was developed referring to previous studies^18^,^53^. Each pixel is 7.5 μm × 7.5 μm, and the number of pixels is 120 × 268, which corresponds to an imaging area of 900 μm × 2,010 μm (Fig. 1b). The sensor chip size is 1 mm × 3.5 mm. The CMOS image sensor and LEDs are arranged with wire-bonding on an atraumatic flexible polyimide circuit, which was newly made to drive dual LEDs. The blue and green LEDs (chip size: 280 μm × 305 μm, peak wavelength: 470–490 nm, 525–545 nm, maximum intensity: 90 mcd, 240 mcd, Epistar Corp., Taiwan) were arranged near the sensor chip for photostimulation and excitation. Eight green LEDs, which are connected to a circuit in parallel, can be turned on synchronously, whereas three blue LEDs, which are connected to a circuit with three independent anodes and one shared cathode, can be lighted individually. To pass the fluorescence and intercept the stimulation and excitation light, a red absorption filter (long-pass liquid photoresist filter: >600 nm, FUJIFILM Corp., Japan) was superimposed on the pixel array using a spin-coating, baking and UV cross-linking process. The filter thickness is approximately 1 μm. The entire sensor was covered by a waterproof resin. The analog output data stream from the sensor was transferred by a newly developed transit board, which has a low-noise operational amplifier (ADA4898-1, Analog Devices, USA). The pre-amplified data were converted to 12-bit digital data by a custom-developed control board, and the digital data were processed and displayed as 10-bit pseudo-colored images by custom-developed software. One computer was used to control the sensor and record and store the image data. The printed circuit boards were designed using computer-aided design (Altium designer, Altium, USA).

Concerning the photostimulation, 25 mcd μLED can activate the neural cells in the brain, which was evaluated with electrophysiological experiment^54^. Concerning the fluoroimaging, our imaging device can visualize the activities of the neural cells in the brain by fluorescence voltage sensitive dye imaging, which was evaluated in comparison with the electrophysiological experiment^24^,^25^.

Although a waterproof, long-term, stable drive in an imaging system similar to that considered herein has been reported in a previous study, when the neural cells were cultured on the LSI chip^22^, all of the newly developed devices were immersed in physiological saline and were checked by imaging fluorescent beads that were similar in size to a cell, in order to verify its waterproof characteristics and cellular resolving power beforehand.

### Three-dimensional (3D) cell culture on the LSI chip

**Figure 3:**
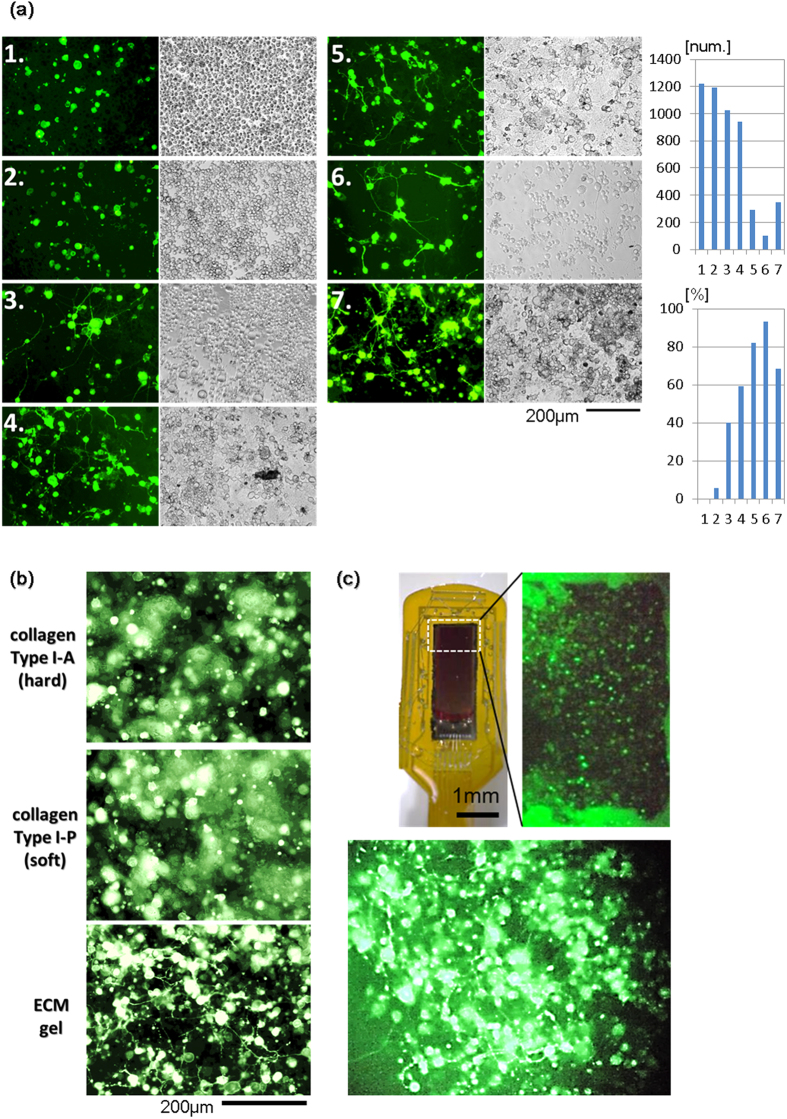
Requirement study of the 3D culture condition of Neuro2a cells with collagen-gel or matrix-gel, on the dish or on-chip. (**a**) Neuro2a cells transfected with green fluorescent protein gene were cultured with seven different media in a 24-well plate, and images were taken using an inverted fluorescence microscope. After culture for 5 days, the proliferated cells and the cells that had neurite-like structures were counted; totals are plotted in the graph. (**b**) The transfected Neuro2a cells were cultured three-dimensionally in three different gels in a glass bottom dish. After culture for 5 days, images were taken using an inverted fluorescence microscope. (**c**) Neuro2a cells were cultured in ECM gel on the sensor chip. After culture for 5 days, images were taken using a stereo fluorescence microscope. The upper image is magnified the under image.

**Figure 4:**
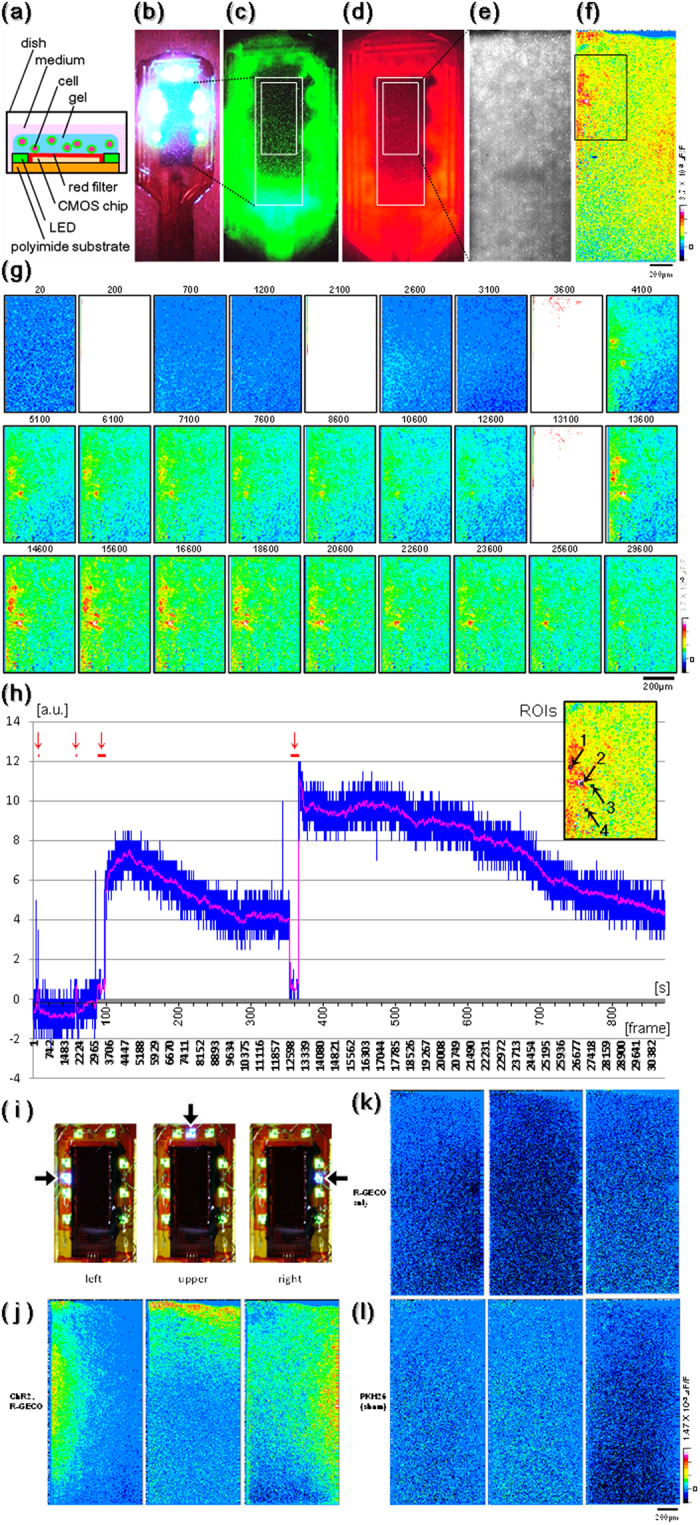
(**a**) Schematic diagram of the *in vitro* experiment. The image of the three-dimensionally cultured cells on the sensor was taken using a stereo microscope. (**b**) Bright-field, (**c**,**d**) fluorescence images. (**e**) Fluorescence image taken using the sensor. (**f**) After photostimulation, the change in fluorescence intensity is indicated as a pseudo-colored image. (**g**) Time-lapse images of the area indicated by the black square in (**f**). The numbers at the top of each time-lapse image indicate the number of frame. (**h**) Graph indicating the mean of the fluorescence intensity in the region of interest (red bars and arrows = photostimulation). (**i**–**l**) Experiment involving local photostimulation. The cells were stimulated locally by LEDs in different locations, as shown in (**i**). The cells were transfected with ChR2 and R-GECO1 (**j**) and R-GECO1 (**k**) and were stained with PKH26 (**l**). After photostimulation, the change in fluorescence intensity is indicated as a pseudo-colored image.

**Figure 5:**
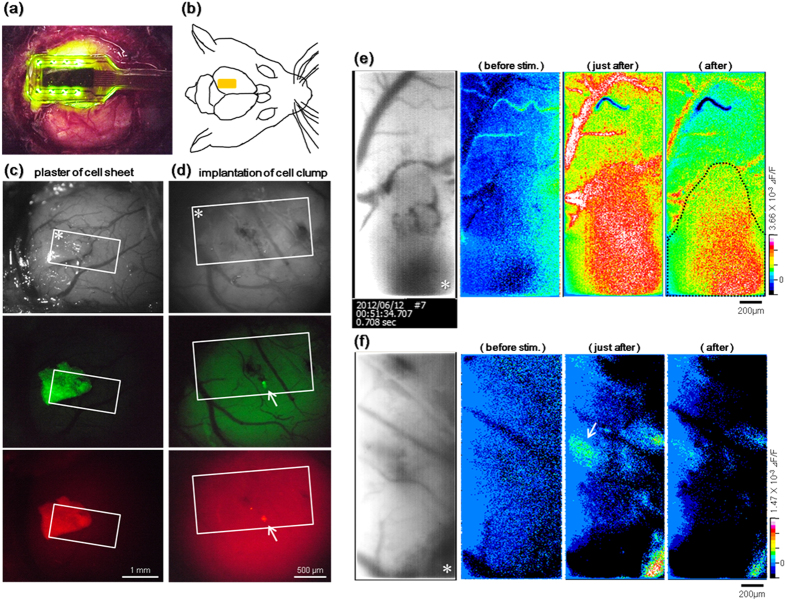
Transplant evaluation examination using the device was performed on the mouse brain. (**a**,**b**) The device was applied to the cortex after craniotomy. (**b**) shows the schematic image of (**a**), and the yellow rectangle in (**b**) indicates the position of the sensor. (**c**,**d**) A gel sheet of three-dimensionally cultured cells was inserted under the dura and plastered to the cortex (**c**). The cell clump implanted into the cortex (white arrow) (**d**). Images taken using a fluorescence stereo microscope. The white rectangles indicate the sensor position. (**e**,**f**) Changes in fluorescence intensity in (**c**,**d**) are indicated as pseudo-colored images. The white asterisks in (**c**–**f**) indicate the same position.

**Figure 6:**
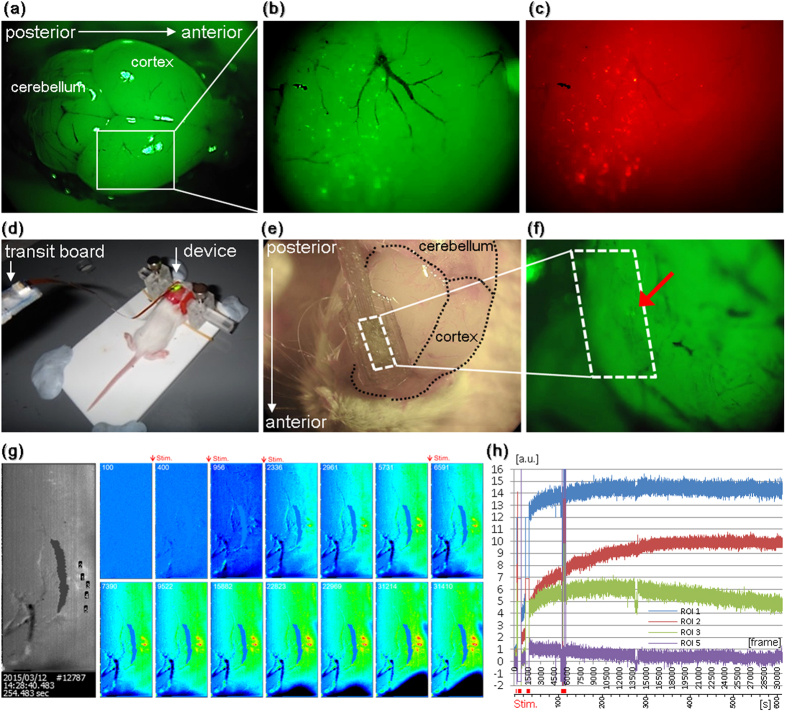
Confirmation of bidirectional, optical communication in the mouse brain. Using in utero electroporation, both ChR2-Venus and O-GECO1 were delivered to the neural cells in the mouse brain. (**a**–**c**) The postnatal day 14 mouse brain was examined for expression of green and red fluorescent proteins. Fluorescence images were taken using a stereo fluorescence microscope. (**d**) shows the experimental setup for application of the device. (**e**,**f**) Bright-field and fluorescence images were taken using a stereo fluorescence microscope. Red arrow shows the presence of fluorescent cells. (**g**) Fluorescence image taken using the sensor. The numbers in the image indicate ROIs. Temporal changes in fluorescence intensity when the brain was stimulated with local blue light are shown as pseudo-colored images. Values from the sequential ROIs are plotted in the graph (**h**). ROIs 4 and 5 do not include positive cells, and were considered as baseline and negative control, respectively.

Neuro2a cells, derived from mouse neuroblastoma cells^55^,^56^, were used as a model of the neural cells. Neuro2a cells were transfected with both a photosensitive cation channel protein (ChR2^57^,^58^) and a red-fluorescent-protein-based genetically encoded Ca^2+^ indicator (R-GECO1^6^) using X-tremeGENE transfection reagents (Roche Applied Science, Germany). These gene expression plasmids were obtained from Addgene (ChR2 #15753, R-GECO1 #32444). The transfected cells were embedded within a collagen gel (Cellmatrix Type I-A, I-P, Nitta Gelatin Inc., Japan) or an extracellular matrix gel (E1270, Sigma-Aldrich, USA) in a manner similar to that previously described^37^, and the liquid gel was pasted on the sensor, which was placed in a dish (Figs 3b and 4a). After gel solidification by warming at 37°C, the sensor was then immersed in Dulbecco’s Modified Eagle’s Medium (DMEM) containing 10% fetal bovine serum (FBS) and antibiotics, and was cultured at 37 °C and 5% CO_2_ in an incubator.

### A drug exposure evaluation system for the micro-sensor

The schematic image shows a drug exposure evaluation system in Fig. S4a, which was newly developed to analyze the physiological activity of on-chip cultured cells. The superjacent liquid level on the sensor does not change because the cell culture compartment is sealed by a cover glass; therefore, accurate, quantitative optical measurements can be performed efficiently. In this experiment, images with a top-down view of the device were taken using a lab-made fluorescence stereo-microscope system with Coolpix 7100. The system was produced using a cheap, generic stereo microscope in combination with LEDs for excitation, and color cellophanes as an absorption filter to pass emitted fluorescence were purchased from a local dollar store. The filter unit was constructed by putting cellophanes in a frame made with chopsticks. The appropriate usage and suitable combination of LED and filter were considered based on the predefined specification of the cellophane, which was analyzed using a hand-made spectrometer. The spectrometer was assembled with the internal parts of a web camera, a slit of paper, and a part of a DVD disk as a diffraction grating. Finally, the discrimination ability of the lab-made fluorescence stereo-microscope system for the fluorescence imaging was verified by actually imaging the fluorescent beads and cells transfected with fluorescent proteins.

## Additional Information

**How to cite this article**: Kobayashi, T. *et al.* “Optical communication with brain cells by means of an implanted duplex micro-device with optogenetics and Ca^2+^ fluoroimaging”. *Sci. Rep.* **6**, 21247; doi: 10.1038/srep21247 (2016).

## Supplementary Material

Supplementary Information
