# Supplementary material for: “Optical communication with brain cells by means of an implanted duplex micro-device with optogenetics and Ca2+ fluoroimaging”
Source: Sci Rep. 2016 Feb 16;6:21247. doi: 10.1038/srep21247 (PMC4754641; doi:10.1038/srep21247)
Supplement: Supplementary Information [file srep21247-s1.doc]

**Supplementary information**

**“Optical communication with brain cells by means of an implanted duplex micro-device with optogenetics and Ca^2+^ fluoroimaging”**

Takuma Kobayashi, Makito Haruta, Kiyotaka Sasagawa, Miho Matsumata, Kawori Eizumi, Chikara Kitsumoto, Mayumi Motoyama, Yasuyo Maezawa, Yasumi Ohta, Toshihiko Noda, Takashi Tokuda, Yasuyuki Ishikawa, and Jun Ohta

**1. Supplementary figures and figure legends:**


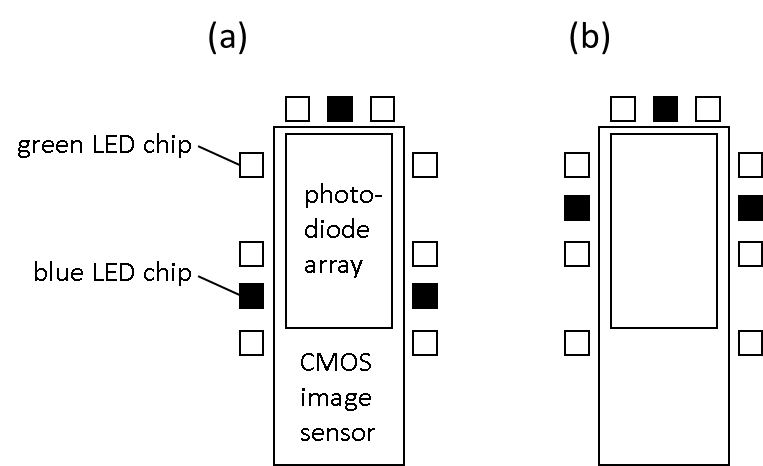


Figure S1. Two variations of the device of which the position of the blue LED chip was different were developed.


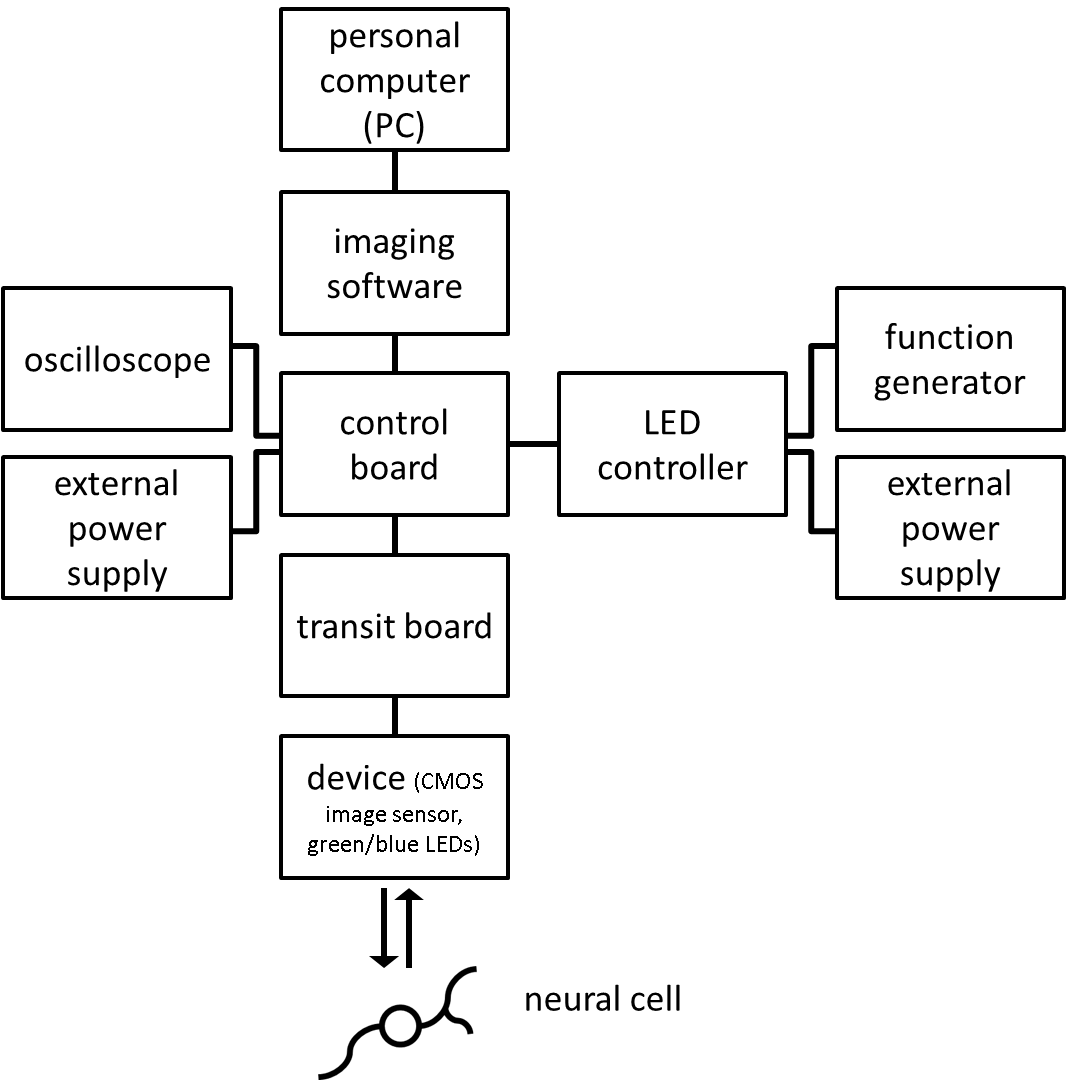


Figure S2. The paradigm of the experimental process is described below.

1. Turn on and start the PC, lab-made control board, lab-made transit board, CMOS imaging sensor, and green LEDs for fluorescence imaging.

2. Adjust green LEDs power appropriately while watching the fluorescence raw/subtracted images that are displayed on the PC screen by the lab-made imaging software.

3. Adjust the signal transduction of the image sensor using the control board for the noise reduction while watching the oscilloscope.

4. Select to turn on blue LED/s for optical stimulation by the lab-made LED controller, and set the function generator to turn on blue LED/s.

5. Start the storage of the imaging data on the PC by the imaging software.

6. First optical stimulation is applied to the neural cells by blue LED, and the reaction of the cell by the stimulation is visualized by the Ca^2+^ imaging. Experimenter can observe the process of the cell response and evaluate if the first stimulation is weak or strong (e.g. the pulse duration, the interval time of the pulse, and total stimulation time are short or long, or voltage is low or high) in real-time.

7. After the real-time evaluation, experimenter can change the setting of the function generator at once, and then, apply the second optical stimulation at the timing when the fluorescent intensity (that means cell activity) returns near to the basement value while watching the PC monitor in real-time.

8. Repeatedly, the experimenter can judge whether the second stimulation is appropriate or not from the continuous Ca^2+^ imaging in real-time.

9. In this way, the experimenter can reply to give the appropriate answer (stimulation) repeatedly for the cell in real-time on demand from the cell which shows its physiological intracellular Ca^2+^ dynamic condition. That is the communication process to the cell.


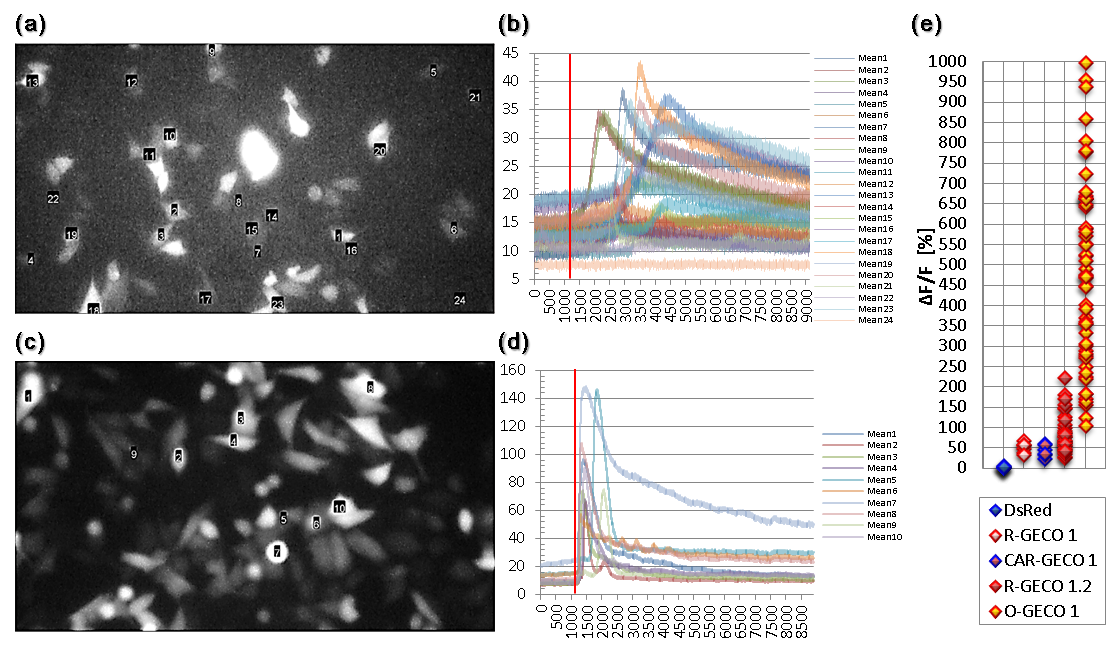


Figure S3. Comparison of the rate of change for several red fluorescent Ca^2+^ indicator genes

Red genetically encoded Ca^2+^ indicators were obtained from Addgene (CAR-GECO1 #45493, R-GECO1.2 #45494, O-GECO1 #46025), and the rates of change in their fluorescence intensity were compared to R-GECO1 with in vitro experiments. HeLa cells cultured in DMEM containing 10% FBS and antibiotics in an incubator at 37°C and 5% CO_2_ were transfected with R-GECO1 (A, B) or O-GECO1 (C, D) using Lipofectamine 2000 regent (Life Technologies, Inc., USA) in a plastic 24-well plate. After culture for 2 days, the transfected cells were stimulated by the application of the histamine solution using a previously described method^s1^. The fluorescence images were taken using a routine inverted fluorescence microscope (Axiovert25, Zeiss, Germany) with a commercial digital camera (Coolpix 7100, Nikon, Japan) (A, C). The numbers in (A, C) indicate ROIs. The change in fluorescence intensity in each ROI in (A, C) are plotted in each graph (B, D), respectively. The sampling rate is 23 frames / seconds (B, D). The red line in B and D indicates the addition of histamine. After the addition of histamine, the fluorescence intensities of each ROI transiently increased, and some of them showed an oscillatory reaction. (E) This graph shows the temporal change in fluorescence intensity (dF/F) of individual cells that were transfected with five different red fluorescent genes. These results indicate that O-GECO1 had the maximum dF/F, which is approximately 1000%.


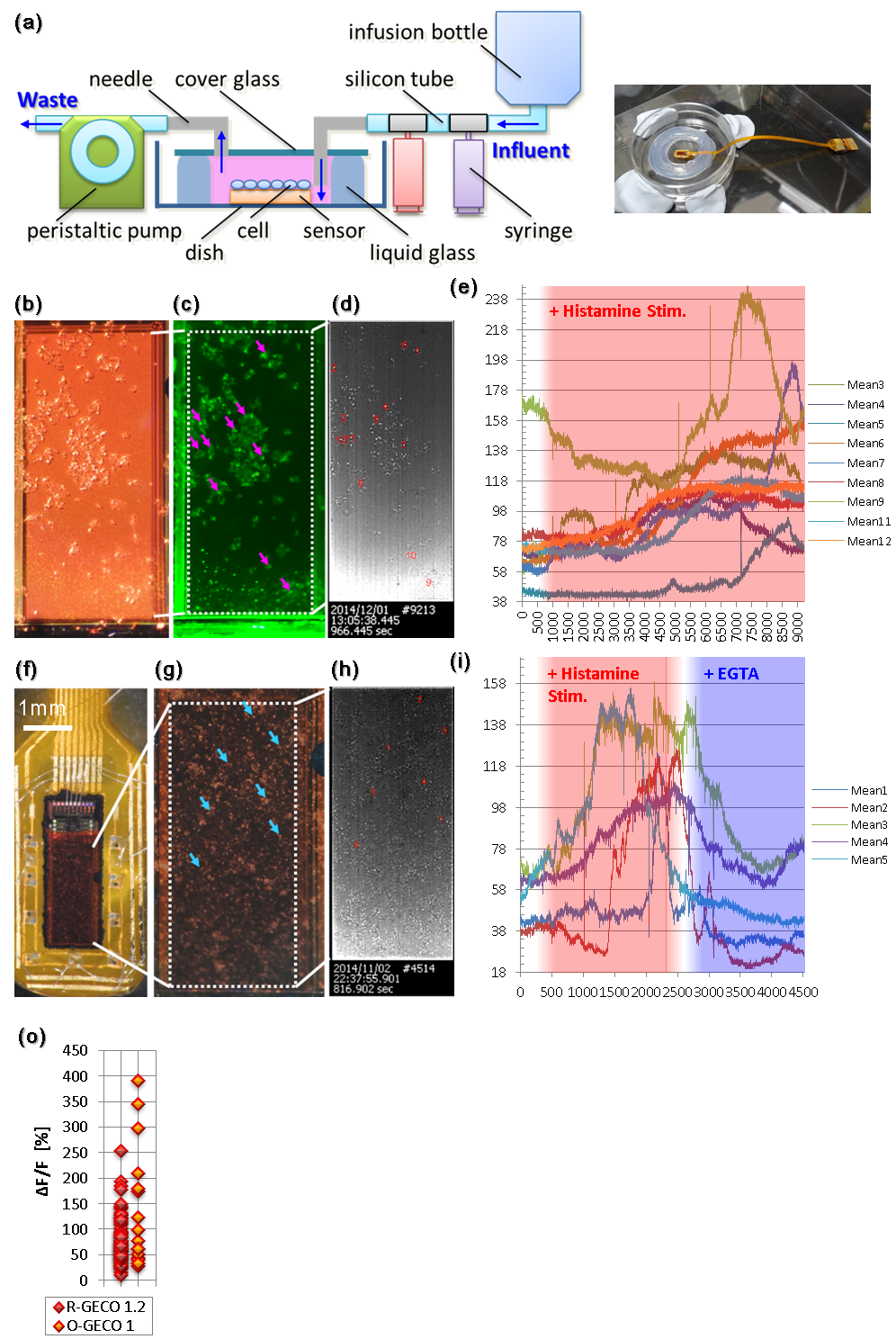


Figure S4. Pharmaceutical transfusion analysis with fluorescence imaging by the sensor

(A) The schematic image shows a drug exposure evaluation system. HeLa cells co-transfected with EGFP and R-GECO1.2 (B–E) or O-GECO1 (F–I) were cultured on the sensor. Bright-field (B, F) and fluorescence images (C, G) were taken using the lab-made fluorescence stereo-microscope system. (D, H) Fluorescence images were taken using the sensor. The numbers in (D) and (H) indicate the ROIs, which are the same position as the selected cells [arrows in (C) and (G), respectively]. (E, I) The graph shows the temporal change in fluorescence intensity in each of the ROIs selected. The sampling rate is 10 frames / seconds. The cells were treated with histamine (E), or with histamine and continuous application of EGTA solution (I). The fluorescence intensity gradually increased with the addition of histamine, but decreased with EGTA administration. These results indicate that fluorescence intensity changes of both R-GECO1.2 and O-GECO depend on the kinetics of the intracellular Ca^2+^ density change, which can be monitored using our sensor. (O) This graph shows the rate of change in fluorescence intensity (dF/F) of individual cells transfected with two different red fluorescent genes. The sensor was able to detect O-GECO1 better than R-GECO1.2.

**2. Supplementary table:**

Table S1. All variations of the culture media in Fig.2 are shown in the table.

**References**

s1. Wu, J. *et al*. Improved orange and red Ca²± indicators and photophysical considerations for optogenetic applications. *ACS Chem. Neurosci.* **4(6)**, 963-972 (2013).
